# Bicycle Helmets and Bicycle-Related Traumatic Brain Injury in the Netherlands

**DOI:** 10.1089/neur.2020.0010

**Published:** 2020-11-17

**Authors:** Crispijn L. van den Brand, Lennard B. Karger, Susanne T.M. Nijman, Huib Valkenberg, Korné Jellema

**Affiliations:** ^1^ Department of Emergency Medicine, Haaglanden Medical Center, The Hague, the Netherlands.; ^4^ Department of Neurology, Haaglanden Medical Center, The Hague, the Netherlands.; ^2^ Department of Emergency Medicine, Treant Zorggroep, Hoogeveen, the Netherlands.; ^3^ Consumer Safety Institute (VeiligheidNL), Amsterdam, the Netherlands.

**Keywords:** bicycle helmets, neurotrauma, trauma, traumatic brain injury

## Abstract

The aim of this study was to determine the association between bicycle helmet use in adults (16 years and older) and traumatic brain injury (TBI) in emergency departments (EDs) in the Netherlands.The conducted research was a retrospective case-control study in patients aged 16 years and older who sustained a bicycle accident and therefore visited the EDs of participating hospitals throughout 2016. Cases were patients with TBI; controls were patients without TBI but with other trauma. Exposure was defined as helmet wearing during the accident. In total, 2133 patients were included in the study, 361 case patients and 1772 controls. Within the TBI group (cases) 3.9% of patients wore a helmet compared with 7.7% of patients in the control (non-head injury) group (odds ratio [OR] 0.49, 95% confidence interval [CI]: 0.28-0.86). No difference in helmet wearing was observed in patients who sustained accidents that involved motorized vehicles (OR 0.91; 95% CI: 0.29-2.83). In conclusion, adult patients (≥16 years of age) with TBI had a significantly lower odds of wearing a bicycle helmet than adult patients with other trauma, adding more evidence that wearing a bicycle helmet effectively protects against TBI.

## Introduction

Worldwide, cycling is a popular form of recreation and a cheap and environmentally friendly mode of transportation. In the Netherlands, a small and densely populated country, cycling is very popular. In fact, the Netherlands could be called the number one cycling country in the world. An average of approximately 900 kilometers per inhabitant is cycled each year, by far the most in the world and about 20 times as much as in the United States.^1–3^ This translates to 27% of all trips in the Netherlands being done by bicycle, again more than in any other country in the world.^4^

Cycling is also a relatively safe mode of transportation compared with other modes of transportation and the health benefits of cycling are substantially higher than the risks associated with cycling.^5,6^ Moreover, there is a correlation between bicycle use in a country and the fatality rate among cyclists. Higher bicycle use in a country is associated with lower fatalities, with the Netherlands having the lowest fatality rate per kilometer cycled.^3^ However, despite investments in road safety and overall decreasing incidence of traffic fatalities, injuries and fatalities among cyclists did not significantly decrease in the last 20 years in the Netherlands. Currently, bicycle accidents are responsible for over 70% of all severely injured traffic participants in the Netherlands.^7^ Severe injury as a result of bicycle accidents has increased by 35% in the last 10 years. Especially in elderly traffic participants this increase is significant even when correcting for aging of the population; one possible explanation is that the elderly do cycle a lot more nowadays than they used to in the past, for example because of the introduction of e-bikes.^7,8^ The rise of traumatic brain injury (TBI) is not only responsible for the growing incidence of persons with bicycle-related injury presenting at the emergency department (ED), but TBI is also the most important cause of death and long-term disability from bicycle injury.^9–13^ Hence, it is crucial to reduce TBI incidence among cyclists.

An obvious way to realize less TBI in cyclists could be by promoting bicycle helmets. However, both public opinion and the scientific literature are divided about bicycle helmets. Some claim that bicycle use decreased after helmets became obligatory in different countries and as a result the health benefits of helmets were negated. For example, bicycle use in New Zealand declined by 51% after it became obligatory to wear a bicycle helmet.^14^ Other authors question whether there is any causality between the decline in cycling and the bicycle helmet law.^15,16^ Regarding the protective value of bicycle helmets, two meta-analyses that included mostly case-control studies both concluded that bicycle helmets reduce serious and fatal head injury by approximately 60–70%.^17,18^ However, other studies question (the magnitude of) this protective effect of bicycle helmets.^19–22^

Although many studies have been conducted to examine the effectiveness of bicycle helmets, remarkably no such study has been performed in the Netherlands. In contrast to other countries, in the Netherlands bicycle helmets are not mandatory or common and bicycle helmet use is fiercely debated.^23–25^

In the current study we examine the association between bicycle helmet use in adults (16 years of age and older) and TBI cases in EDs in the Netherlands.

## Methods

### Data sources and inclusion

In this retrospective case-control study patients aged 16 years and older who sustained a bicycle accident and therefore visited the EDs of participating hospitals throughout 2016 were included using the Dutch Injury Surveillance System (LetselInformatieSysteem; LIS). Cases were defined as patients with TBI who visited the ED of one of the participating hospitals; controls were defined as patients without TBI but with other trauma who visited these EDs. Exposure was defined as (self-reported) helmet-wearing during the accident.

The LIS database is a continuous monitoring system in which in addition to demographics, injury diagnoses and injury mechanisms are registered. LIS is based on 13 geographically distributed EDs in the Netherlands, resulting in a representative 12–15% sample of injury-related ED visits that can be extrapolated to national estimates. For extrapolation of the sample a factor was calculated in which the number of trauma-related ED treatments in LIS hospitals was multiplied by the quotient of all trauma-related hospital admissions in the Netherlands divided by trauma-related hospital admissions in LIS hospitals.^26–29^

To study bicycle-related accidents, extra information was gathered in all LIS hospitals in 2016. Patients who sustained a bicycle accident received a questionnaire within 2 months after their visit to the ED. Patients were asked to complete the questionnaire online or to fill out a paper questionnaire. Ultimately, a sensitivity analysis was conducted in which the study population was corrected for selective (non-)response by a weighing factor, using the age and gender distribution from the total patient population for bicycle accident-related ED treatments from the LIS database.

The study was submitted to the medical ethics review committee (reference number W16_151#16.175), which concluded that the Medical Research Involving Human Subjects Act (WMO) was not applicable. Therefore, official approval of this study by the medical ethics review committee was not required.

### Exclusion

All participants who were not driving on public roads (i.e., parcourse, dirt track, private property) were excluded. Because we focussed on the risk of TBI in normal traffic, we also excluded cyclists who were travelling at a self-reported speed of 25 km/h or more.

Patients with *isolated* injury to the eyeball and/or to the scalp were excluded from the control group because helmet wear possibly protects against these injuries. Patients with a combination of injuries that included scalp or eyeball injury were not excluded from the study.

### Statistical analysis

Data were analysed using descriptive statistics, χ^2^ tests, and Mann-Whitney U tests where appropriate. A significance threshold was set at *p* < 0.05. The Statistical Package for the Social Sciences (IBM Corp., IBM SPSS Statistics for Windows, version 22.0, Armonk, NY, USA) was used for statistical analysis.

## Results

**FIG. 1.**
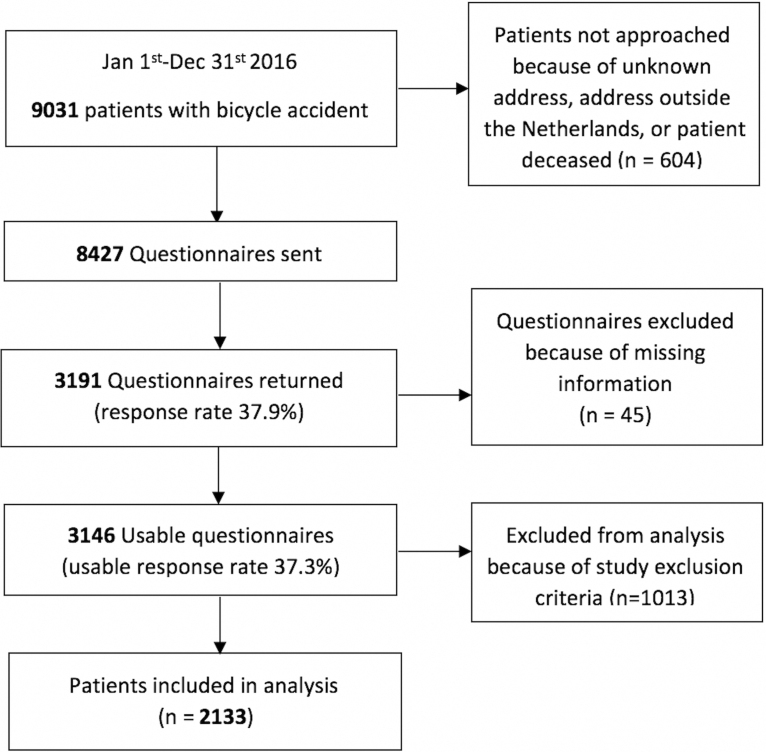
Overview of cases included in the analysis.

Between January 1, 2016 and December 31, 2016, 9013 patients were treated for a bicycle accident in the ED of participating LIS hospitals. Of these 9013 patients 3146 returned a usable questionnaire. After exclusion of patients under 16 years of age, or with other exclusion criteria, 2133 patients were included in the analysis (Fig. 1). These 2133 patients were 361 cases (patients with TBI) and 1772 controls (patients without TBI). Of the entire group 60.4% were female. The mean age was 58.5 years. To assess comparability of cases (patients with TBI) and controls (patients without TBI), patients *without* helmet wear were compared between cases and controls. It appeared that patients with TBI were more often male than controls; no other significant differences were observed between cases and controls (Table 1).

**Table 1A.** Baseline Characteristics of Cases and Controls, All Patients

	Cases (TBI),* n* = 361	Controls (non-TBI),* n* = 1772	Missing	P-value
Age, years (mean)	58.7	58.5	0	0.63
Male sex (*n*, %)	161 (44.6%)	684 (38.6%)	0	0.03
Helmet wear (*n*, %)	14 (3.9%)^a^	135 (7.7%)^a^	16 (0.8%)	0.03
Motorized vehicle collision (*n*, %)	70 (40.9%)^b^	242 (32.4%)^b^	1214 (56.9%)^d^	0.03
Bicycle type (*n*, %)			26 (1.2%)	
Commuter bicycle	205 (57.7%)^c^	951 (54.3%)^c^		0.23
Mountain bike	8 (2.3%)^c^	46 (2.6%)^c^		0.69
Racing bike	18 (5.1%)^c^	125 (7.1%)^c^		0.16
Bike with pedal support	117 (33.0%)^c^	602 (34.4%)^c^		0.61
Other	7 (2.0%)^c^	28 (1.6%)^c^		0.62

^a^ Unknowns and missings (for helmet wear) are excluded: cases (TBI), *n* = 359; controls (non-TBI), *n* = 1758.

^b^ Unknowns and missings (for cause of accident) are excluded: cases (TBI), *n* = 171; controls (non-TBI), *n* = 748.

^c^ Unknowns and missings (for bike types) are excluded: cases (TBI), *n* = 355, controls (non-TBI), *n* = 1752.

^d^ The high number of missings is probably caused by the nature of the question, “What did you collide with?” In many cases this was unknown or not applicable.

TBI, traumatic brain injury.

**Table 1B.** Baseline Characteristics of Cases and Controls, Patients without Helmet Wear Only

	Cases (TBI),* n* = 345	Controls (non-TBI),* n* = 1623	Missing	P-value
Age, years (mean)	59.0	58.7	0	0.71
Male sex (*n*, %)	149 (43.2%)	566 (34.9%)	0	<0.01
Motorized vehicle collision (*n*, %)	66 (40.5%)^a^	225 (33.4%)^a^	1131 (57.5%)^c^	0.09
Bicycle type (*n*, %)			26 (1.2%)	
Commuter bicycle	202 (59.6%)^b^	935 (58.3%)^b^		0.67
Mountain bike	7 (2.1%)^b^	23 (1.4%)^b^		0.39
Racing bike	6 (1.8%)^b^	31 (1.9%)^b^		0.84
Bike with pedal support	117 (34.5%)^b^	589 (36.7%)^b^		0.44
Other	7 (2.1%)^b^	25 (1.6%)		0.51

^a^ Unknowns and missings (for cause of accident) are excluded: cases (TBI), *n* = 163; controls (non-TBI), *n* = 674.

^b^ Unknowns and missings (for bicycle types) are excluded: cases (TBI), *n* = 339, controls (non-TBI), *n* = 1603.

^c^ The high number of missings is probably caused by the nature of the question “What did you collide with?” In many cases this was unknown or not applicable.

TBI, traumatic brain injury.

Within the TBI group (cases) 3.9% of patients wore a helmet compared with 7.7% of patients in the control (non-TBI) group (odds ratio [OR] 0.49, 95% confidence interval [CI]: 0.28-0.86). These differences were clearly visible in patients with accidents that did not involve motorized vehicles (OR 0.27; 95% CI: 0.08-0.87). In contrast, in patients with accidents that involved motorized vehicles no difference was found between the groups (OR 0.91; 95% CI: 0.29-2.83; Table 2).

**Table 2.** Odds for Traumatic Brain Injury in Cyclists

	Odds ratio	95% confidence interval		P-value
Odds for TBI wearing a helmet	0.49	0.28	0.86	0.01
Odds for TBI in a motorized vehicle collision wearing a helmet	0.91	0.29	2.83	0.87
Odds for TBI in an accident without motorized vehicle wearing a helmet	0.27	0.08	0.87	0.03
Odds for mild traumatic brain injury wearing a helmet	0.47	0.25	0.88	0.02
Odds for severe traumatic brain injury wearing a helmet	0.54	0.17	1.74	0.30

TBI, traumatic brain injury.

For all different types of bicycles patients were less likely to have worn a helmet in the TBI group compared with the control (other injury) group. However, this difference did not reach statistical significance in any of the bicycle types (Table 3).

**Table 3.** Odds for Traumatic Brain Injury per Bicycle Type (Helmet Wearing vs. Not Helmet Wearing)

	Odds ratio	95% confidence interval		P-value
Commuter bicycle	0.58	0.07	4.65	0.61
Mountain bike	0.15	0.02	1.32	0.09
Racing bike	0.67	0.23	1.93	0.45
Bike with pedal support	0.26	0.02	4.57	0.36

A sensitivity analysis was performed to correct for selective (non)-response to the questionnaire. This additional analysis did not essentially change the results of the study. The odds of wearing a bicycle helmet in TBI compared with other trauma was 0.52 (95% CI: 0.29-0.94) in the sensitivity analysis.

## Discussion

Adult patients (≥16 years of age) who presented to the ED with TBI wore a bicycle helmet significantly less often than adult patients who presented with other trauma. Therefore, wearing a bicycle helmet appears to effectively protect against TBI. However, when focusing on adult cyclists who experienced a motorized vehicle collision (MVC) we found no indication for a reduced risk of TBI because of bicycle helmet use.

In recent years there has been a fierce discussion about the use, active promotion, or even obligation of bicycle helmets. On one side of the spectrum are the promotors of bicycle helmets who claim that it is a good way to halt the growing incidence of bicycle-related TBI, especially in vulnerable groups such as children and elderly.^22,25,30–32^ On the other end of the spectrum there is fierce opposition to active promotion or obligatory use of bicycle helmets. Opponents of (obligatory) helmet use doubt the protection offered by helmets and fear that obligatory helmet use will lead to decline in cycling.^13,24,33,34^

In our control group 7.7% of patients wore a helmet, which is comparable to results of a survey conducted in 2008, in which 7.5% of all cyclists with a self-reported speed of less than 25 km/h without head injury wore a helmet (unpublished data, obtained from NLVeiligheid).^35^ In our control group helmet use is still very infrequent on commuter bicycles (0.8%), but high on racing bikes (75%) and mountain bikes (49%). These results are all comparable to those in the 2008 survey.

The results of this study appear to show that helmet use in cyclists reduces the risk of TBI. However, the case-control design of the study makes it impossible to draw firm conclusions regarding a causal relationship or magnitude of this relationship. Opponents of this theory point out that another explanation for the observed OR is that cyclists with helmets are more often sports cyclists (mountain bikers and racing cyclists), who according to some might have relatively more non-TBI trauma than other cyclists.^36^ Our results do not support this explanation, as the ratio between TBI and non-TBI was not different for (non-helmet wearing) sports cyclists compared with those on normal bicycles.

We found no significant relationship between bicycle helmet use and (reduced) risk of TBI when bicyclists were involved in MVCs. This could be explained by the fact that bicycle helmets are designed to protect against an impact of approximately 20 km/h; in most MVCs the impact is likely to be (much) higher.^37^ The assumed larger protective effect in one-sided bicycle crashes compared with bicycle-MVCs is in line with an earlier study.^38^ However, this does not have to discredit the bicycle helmet use because motorized vehicles were involved in a minority of TBIs in our study. This is in line with other research on this subject that also shows that in the majority of the patients with TBI no motorized vehicles were involved.^25^

### Strengths and limitations

The current study is the first study of its kind in the Netherlands. Strengths of the study are the large number of participants and the detailed information obtained. Limitations of our study include the lack of exact information about bicycle helmet use in the Netherlands in non-injured cyclists. Therefore, we used patients who presented to the ED without head injury as a control group as we had exact information about helmet use in that group. In addition, bicycle helmet use in our study (7.7% in the control group) was comparable to a survey in 2008 that showed bicycle helmet use of 7.5% in patients without head injury.^35^ Another related limitation is the case-control design of the study; therefore only association and no causal relationship between helmet use and TBI can be proven. Also, the response rate of 37% is an additional limitation of this study. The primary analysis was conducted using the unweighted results, hence not corrected for selective non-response. Therefore, these results may not be representative for the entire LIS population. To take this into account a sensitivity analysis, corrected for selective non-response in certain demographic groups, was also performed. Possible selective non-response based on injury severity is not known and could not be corrected for. However, we have no indication that this affected patients with or without helmets unevenly.

## Conclusion

In this study we found that patients with TBI due to bicycle accidents did not wear helmets as often as a comparable control group. This association could not be established for patients with TBI as a result of a collision between a bicycle and a motorized vehicle. This study has some limitations, but the results strongly suggest that TBI in adult cyclists could be reduced if cyclists in the Netherlands would wear a helmet more often. Future research should focus on establishing the exact frequency of bicycle helmet use in the Netherlands and ways to promote helmet use without discouraging cycling.

## Abbreviations Used

CI: confidence interval
ED: emergency department
LIS: LetselInformatieSysteem
MVC: motorized vehicle collision
OR: odds ratio
WMO: Medical Research Involving Human Subjects Act
TBI: traumatic brain injury
